# The Molecular Landscape and Biological Alterations Induced by PRAS40-Knockout in Head and Neck Squamous Cell Carcinoma

**DOI:** 10.3389/fonc.2020.565669

**Published:** 2021-01-08

**Authors:** Gang Chen, Zhexuan Li, Changhan Chen, Jiajia Liu, Weiming Zhu, Li She, Huimei Huang, Yuexiang Qin, Guancheng Liu, Juncheng Wang, Yong Liu, Donghai Huang, Qinglai Tang, Xin Zhang, Gangcai Zhu

**Affiliations:** ^1^ Department of Otolaryngology-Head and Neck Surgery, The Xiangya Hospital, Central South University, Changsha, China; ^2^ Department of Otolaryngology-Head and Neck Surgery, The Second Xiangya Hospital, Central South University, Changsha, China; ^3^ Fuzhou Medical College of Nanchang University, Fuzhou, China; ^4^ Otolaryngology Major Disease Research Key Laboratory of Hunan Province, Changsha, China; ^5^ Clinical Research Center for Pharyngolaryngeal Diseases and Voice Disorders in Hunan Province, Changsha, China

**Keywords:** head and neck squamous cell carcinoma, prolin-rich Akt substrate of 40 kDa, CRISPR/Cas9, metastasis, The Cancer Genome Atlas, immunomicro-environment

## Abstract

PRAS40 (Prolin-rich Akt substrate of 40 kDa) is a critical protein, which directly connects PI3K/Akt and mTORC1 pathway. It plays an indispensable role in the development of various diseases. However, the relationship between PRAS40 and head and neck squamous cell carcinoma (HNSCC) remains unclear. Here, our study indicated that high expression of PRAS40 mRNA is a favorable prognostic factor in HNSCC patients by analyzing 498 clinical and mRNA data. Moreover, we confirmed that CRISPR/Cas9 induced PRAS40-knockout would promote colony formation, cell migration, and invasion in several HNSCC cell lines. RNA-seq was employed to investigate the further possible mechanisms involving the above regulations by PRAS40 in HNSCC cells. The molecular landscape contributed by 253 differentially expressed mRNA after PRAS40-knockout was enriched in TGF-beta, PI3K-Akt, P53, mTOR, NF-*κ*B signaling pathway. Partial molecular alternations within these pathways were validated by qPCR or Western blotting. Besides, we found that high expression of PRAS40 in HNSC patients would present more CD8^+^ T and T follicular helper cells, but less Th17 cells than the patients with low expression of PRAS40. The altered molecular pathways and tumor-infiltrating immune cells might associate with the mechanism of PRAS40 being a suppressor in HNSCC cells, which would provide a potential prognostic predictor and therapeutic target in HNSCC patients.

## Introduction

The head and neck squamous cell carcinoma (HNSCC) is the sixth most prevalent cancer worldwide (1), with an incidence of approximately 650,000 new patients annually (2). Although the diagnosis methods and therapeutic arsenal have been refined and improved in recent decades, the overall 5-year survival rate of HNSCC patients has been holding around 60% (3). Tumor metastasis and uncontrolled growth are the main reasons for the unsatisfied survival rate in HNSCC patients (4, 5). Tumor metastasis is a highly regulated and complicated process composed of local invasion, intravasation, extravasation, and metastatic colonization at distant sites (6). Abundant evidence has demonstrated that the Akt and mTOR pathway activations are critical to cancer growth, metastasis, and resistance to therapy (7, 8). Due to PIK3CA mutations or other growth stimuli, the constitutive activation of PI3K/AKT/mTOR signaling was observed in more than 90% of HNSCC patients (9). The drugs targeting AKT or mTOR pathway, such as MK2206 or Temsirolimus, presented a promising effect to improve the prognosis of HNSCC patients (10). However, the tumor would develop quick resistance to these targeted therapies in several ways. Identifying novel targets or a dual inhibitor of AKT and mTOR pathway may provide potential support for treating HNSCC patients.

PRAS40, a 40 kDa proline-rich Akt substrate protein, locates at 19q13.33 and functions as an intersection in PI3K/Akt and mTOR pathway. PRAS40 was initially discovered to be a phosphorylated Akt kinase substrate protein (11), which was also named AKT1S1. Subsequently, it was found as a constrained component of mTORC1 (12). Akt could phosphorylate the amino acid residue of PRAS40 on Thr246/198 or Ser183/221/88/92/116/202/203/211, mTORC1, insulin, and PDGF, which could relieve the restrained activity of mTORC1 (12–15). Besides, PRAS40 could interact with ribosomal protein L11 (RPL11) and modulate P53 expression in turn (16). Our previous study showed that PRAS40 could regulate NF-*κ*B transcription activity through physical association with p65 (17). Given the vital effects of mTORC1, P53, and NF-*κ*B on cells, PRAS40 would be expected to play a crucial role in tumor initiation and development.

However, the reports about PRAS40 in tumors seemed controversial. On the one hand, PRAS40 was considered as an oncogene in melanoma, prostate cancer, liver cancer, and Ewing sarcoma by PRAS40-knockdown investigations (18–20). On the other hand, PRAS40 could suppress tumor growth in gallbladder cancer, cervical squamous cell cancer, and colon cancer (21, 22). As far as we are aware, there is no study regarding the role of PRAS40 in head and neck cancer. Here, our study found that high expression of PRAS40 mRNA is a favorable prognosis factor in HNSCC patients. Moreover, we confirmed that CRISPR/Cas9 induced PRAS40-knockout would promote cell colony formation, migration, and invasion in several HNSCC cell lines. RNA-seq was employed to investigate the further possible mechanisms involving the above regulations by PRAS40 in HNSCC cells. The molecular landscape contributed by 253 differential expressed mRNAs after PRAS40-knockout was enriched in TGF-beta, PI3K-Akt, P53, mTOR, NF-*κ*B signaling pathway. Partial molecular alternations within these pathways were validated by PCR or western blot. Besides, we found that high expression of PRAS40 in HNSC patients would present more CD8^+^ T and T follicular helper cells, but less Th17 cells than the patients with low expression of PRAS40. The altered molecular pathways and tumor-infiltrating immune cells might associate with the mechanism of PRAS40 being a suppressor in HNSCC cells, which would provide a potential prognosis predictor and therapeutic target in HNSCC patients.

## Materials and Methods

### TCGA Data Download and Preprocessing

All clinical follow-up and mRNA expression data (including 498 samples) were downloaded from the TCGA datahub (https://portal.gdc.cancer.gov/). The immune cell deconvolution applied to HNSC samples was performed by the CIBERSORT method (23).

### Cell Culture

Dr. Zhuo kindly provided Tu686 cell line, Georgia Chen (Winship Cancer Institute, Emory University School of Medicine, Atlanta, GA, USA) (24). The 6-10B cell line was purchased from the Cell Center of Central South University (Changsha, China). As we described previously (25), Tu686 cells were cultured in DMEM/F12 (1:1) (Cytiva, USA, Cat. No. SH30023.01); 6-10B cells were cultured in RPMI1640 medium (Cytiva, USA, Cat. No. SH30027.01). All media were supplemented with 10% fetal bovine serum (ThermoFisher, USA, Cat. No.16000044), 100 IU/ml penicillin, and 100 μg/ml streptomycin at 37°C with 5% CO2 in an incubator. Cells have been checked to ensure that all in free of contamination and exponential growth so that they were used for subsequent experiments.

### CRISPR/Cas9 Plasmid Construction and Validation

Genomic sequences of human PRAS40 were retrieved from the NCBI Consensus Coding Sequence database (http://www.ncbi.nlm.nih.gov/CCDS/). For each consensus coding sequence (CCDS) entry, exons 2 were included as candidate exons for library design. Next, all possible Cas9 sgRNA sequences of the form (N)20NGG were listed as candidate targets for each candidate exon. Due to the limited laboratory conditions, the synthesis and screening of recombinant plasmids were completed by GeneCopoeia (Guangzhou, China, Cat No. HCP259164-CG04-3-10-a/b/c). The oligonucleotides used to respective sgRNAs were listed in **Table S1**. Enzyme digestion was performed to validate these plasmids.

### The Establishment of PRAS40 Knockout Cell Lines

The knockout of PRAS40 in Tu-686 and 6-10B cell lines was achieved by CRISPR/Case9 system. The sgRNA was pre-designed to the target site in the PRAS40. Briefly, cells were transfected at 50–60% confluency in a 6-well plate with 2 ug CRISPR/Cas9 expression vector and 6 ul Fugene transfection reagent (Promega, USA, Cat. No. E2311). After 48 h, the cells with green fluorescence were sorted by flow cytometry and significantly diluted into a 6-well plate. Seven lines of 6–10B (named as 6-10BPRAS40-KO-1 to 6-10BPRAS40-KO-7) and five lines of Tu686 single clone (labeled as Tu686PRAS40-KO-1 to Tu686PRAS40-KO-5) survived successfully. The PRAS40 protein expression was confirmed as being knockout by western blot (**Supplementary Figure 1**). Three PRAS40 knockout and corresponding wild type cell lines were the only sources used throughout the further functional and mechanistic investigations.

### Colony Formation Assay

Regarding colony formation, a density of 300 cells per well was cultured in the 6-well plate. After 14 days, cells were fixed with 0.1% crystal violet and stained with 4% methanol. Colonies containing more than 50 cells were counted using the Image J software. The average number of colonies was determined from three duplicated wells.

### Wounding-Healing and Transwell Invasion Assay

For wound healing assay, cells were seeded in six-well plates and cultured to reach 90% confluency. Cells were disrupted with a disinfected micropipette tip for 48 h. A microscope captured the images of bruised areas in the wells. These experiments were performed in triplicates.

Transwell chambers were coated with 200 ug/ml of Matrigel (Corning, USA, Cat.No.356255). Cells were seeded on the top of Matrigel at a concentration of 2 × 104 cells/well. After 48 h of culture, the culture media and Matrigel were removed, while the cells that crossed the membrane of the insert were fixed with 0.1% crystal violet and stained with 4% methanol. The average number of cells within three random visions was counted under the light microscope. The experiment was repeated three times.

### Western Blotting Assay

Thirty micrograms of total protein was separated by 10% SDS-PAGE for 1 h. These proteins were transferred to a polyvinylidene difluoride membrane for 90 min (Millipore, USA. Cat. No. IPFL85R). After the membrane was blocked, it was incubated with primary mouse antibodies against PRAS40 (1:1,000 dilution, Proteintech, USA, Cat. No. 21097-1-AP), mTOR (1:1,000 dilution, Abcam, USA, Cat. No. ab109268), p-mTOR (1:1,000 dilution, Abcam, USA, Cat. No. ab109268) or GAPDH (1:1,000 dilution, Proteintech, USA, Cat. No. 1E6D9) at 4°C overnight. After incubation, the membrane was washed with PBST (Phosphate Buffered Saline with Tween-20; Cytiva, USA, Cat. No. SH30256.01) and incubated with HRP-labeled goat anti-rabbit IgG or HRP-labeled goat anti-mouse IgG (1:3,000 dilution, TransGen Biotech, China, Cat.No. HS201-01) for 1 h at room temperature. Finally, the membrane was incubated with the HRP (ThermoFisher, USA, Cat.No.21130), and image the blot using X-ray film (BIO-RAD, USA). Each experiment was repeated in triplicate.

### RNA Sequence and Quantitative Real-Time PCR

According to the manufacturer’s protocol, total RNA extracted from knockout and wild type cells were reverse transcripted to cDNA by All-in-One First-Strand cDNA Synthesis kit (GeneCopoeia, USA). The RNA sequencing service, such as sequencing, read alignment, and DEGseq analysis, was provided by a technical company (Reo Health Inc, China). After cDNA synthesis, quantitative PCR was carried out using All-in-One qPCR Mix (GeneCopoeia, MD, USA) on ABI 7500HT System (Applied Biosystems, Foster City, CA). The primers used in this study were summarized in **Table S1**.

### GO and KEGG Enrichment Analysis

The Gene Ontology (GO) enrichment analysis with the corrected p-value was applied for the differentially expressed genes indicated in the RNA sequencing results. KEGG (Kyoto Encyclopedia of Genes and Genomes) is a database resource for systematic analysis of gene function and genomic information. It helps researchers to study gene and expression information as a whole network (http://www.genome.jp/kegg/). We used an online tool (https://david.ncifcrf.gov/) to test the statistical enrichment of differential expression genes (DEGs) in KEGG pathways.

### Statistical Analyses

All data were evaluated using GraphPad Prism (version 6, San Diego, USA), or R (version 3.6.1). Chi-Squared test or one-way ANOVA (analysis of variance) was performed to assess the significance of differences between two groups of samples. A Dunnett’s test with ANOVA was applied to pairwise comparisons in more than two groups by GraphPad Prism. Categorical variables are expressed as absolute frequencies and percentages, and continuous data were analyzed with descriptive statistics (mean values). The Kaplan–Meier method and Log-rank test were applied for survival analysis, and identification of relevant prognostic factors was performed by univariate and multivariate Cox regression analysis. The best cut-off for PRAS40 high expression was determined by the “survival” package (https://github.com/therneau/survival). P < 0.05 was considered statistically significant. Two-tailed tests were performed. The codes used in this study are available for the asking.

## Result

### The Expression and Clinical Significance of PRAS40 in HNSCC

**Figure 1:**
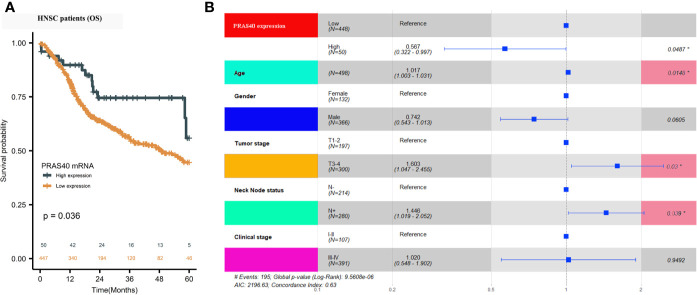
The clinical significance of PRAS40 expression in HNSCC. **(A)** Kaplan-Meier survival analysis implies that five-year overall survival tends to happen in favor of patients with high PRAS40 mRNA expression (p = 0.036). **(B)** Forest plot shows that the different PRAS40 mRNA levels could be an independent factor in HNSCC patients after age, gender, and clinical-stage adjustment (p = 0.0487).

A total of 498 cases of HNSCC patients were included in our analysis. The correlation between the main clinical parameters and PRAS40 expression in HNSCC patients are summarized in **Table 1**. Briefly, the expression of PRAS40 was noticed to have no association with age, gender, tumor stage, node status, and clinical stage in HNSCC patients. As shown in **Figure 1A**, Kaplan–Meier survival analysis implied that the five-year overall survival (OS) tended to favor patients with high PRAS40 expression (p = 0.036). Given the association of PRAS40 expression and survival in HNSCC patients was confirmed, we are led to clarify the independent factors affecting overall survival in these patients. As exhibited in **Table 2**, variables including age, gender, tumor size (T stage), neck nodal metastasis, clinical stage, and PRAS40 expression are significantly associated with survival hazard ratios of 5 years’ death in univariate cox analysis (all p < 0.05). According to the multivariate cox model analysis, PRAS40 expression was identified as independent factors predicting overall survival in HNSCC patients (**Figure 1B**). The hazard ratios of patients with high PRAS40 expression over PRAS40 low expression is 0.567 (0.322–0.997, p = 0.048) after adjusting age, tumor size, neck node metastasis.

**Table 1:** The PRAS40 expression in HNSC patients with different clinical parameters.

Clinical parameters	PRAS40 mRNA expression*		p-value (Chisq test)
	Low	High	
	(n=448)	(n=50)	
**Age(years)**			0.87
Mean (SD)	60.9 (12.1)	61.9 (10.7)	
Median [Min, Max]	61.0 [19.0, 90.0]	60.0 [40.0, 82.0]
**Gender**			0.35
Female	122 (27.2%)	10 (20.0%)	
Male	326 (72.8%)	40 (80.0%)	
**Tumor stage**			0.15
T1-2	172 (38.4%)	25 (50.0%)	
T3-4	275 (61.4%)	25 (50.0%)	
Missing	1 (0.2%)	0 (0%)
**Neck nodal metastasis**			0.34
N-	196 (43.8%)	18 (36.0%)	
N+	248 (55.4%)	32 (64.0%)	
Missing	4 (0.9%)	0 (0%)	
**Distant Metastasis**			1
M0	434 (96.9%)	49 (98.0%)	
M1	4 (0.9%)	0 (0%)	
Missing	10 (2.2%)	1 (2.0%)
**Clinical stage**			1
I-II	96 (21.4%)	11 (22.0%)	
III-IV	352 (78.6%)	39 (78.0%)	

TNM stage is based on the pathologic stage if available in the follow-up data; otherwise, clinic information will be used. Missing means no information reported. *The cutoff value of high PRAS40 expression is 58.29542 (Transcripts Per Kilobase Million, TPM).

**Table 2:** The Significant survival hazard ratios of 5 years’ death in HNSC.

	Univariate cox model	
	HR (95%CI)	p-value
**Age**
Younger vs. Older	0.74 (0.562-0.984)	**0.038**
**Gender**
Male vs. Female	0.74 (0.546-0.996	**0.047**
**Tumor size**	
T3-4 vs. T1-2	1.7 (1.284-2.378)	**<0.001**
**Neck nodal metastasis**
N+ vs. N-	1.4 (1.08-1.931)	**0.013**
**Clinical stage**
III-IV vs. I-II	1.8 (1.199-2.609)	**0.004**
**PRAS40 expression**
High vs. Low	0.55 (0.315-0.971)	**0.039**

The cutoff of age is the median (61 years old) of HNSC patients. The bold values mean p < 0.05, which is considered as significant difference.

### Knockout of PRAS40 Promotes the Colony Formation in HNSCC Cell Lines

**Figure 2:**
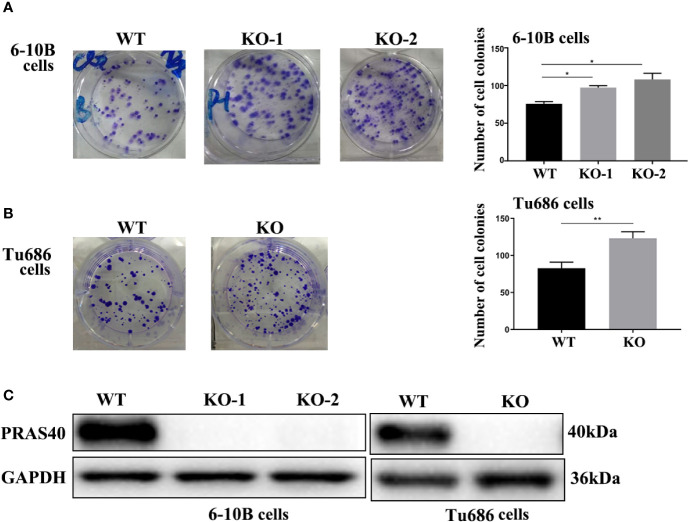
The knockout of PRAS40 promotes colony formation in HNSCC cell lines. **(A, B)** Colony formation assays show that knockout of PRAS40 increases the number of colony formation when comparing to their parent cells (6-10B and Tu686). Quantitative data for triplicated experiments are presented in the right panel. The mean ± SD of cell colonies raises 75 ± 3 to 97 ± 3 and 108 ± 9 in 6-10B cell cohorts (p<0.05). A similar tendency is observed in Tu686 related cell lines (123 ± 9 vs. 82 ± 8, p<0.01). **(C)** PRAS40 proteins are double-checked to confirm there is no PRAS40 expression in our experimental knockout 6-10B and Tu686 cell lines. WT means wildtype. KO means knockout. *p < 0.05 , **p < 0.01.

To confirm the above finding that PRAS40 might confer a longer survival for HNSCC patients, we investigated the potential functions of PRAS40 in multiple HNSCC cell lines. Firstly, we established multiple HNSCC cell lines with PRAS40 knockout by CRISPR/Cas9. The PRAS40 protein expression was confirmed as no expression (**Figure 2C**) in all the knockout cell lines comparing to their parent cells. Compared to the wild type cell lines, the colony formation assays showed cells with PRAS40 knockout would significantly increase colony formation capability. As presented in **Figure 2A**, the mean ± SD of cell colonies raised from 75 ± 3 to 97 ± 3 and 108 ± 9 in 6–10B cell cohorts (p < 0.05). A similar tendency was observed in Tu686 related cell lines (123 ± 9 *vs.* 82 ± 8, p < 0.01, **Figure 2B**). These may indicate that PRAS40 could restrain the capability of HNSCC tumor formation.

### Knockout of PRAS40 Increases the Invasion and Metastasis in HNSCC Cell Lines

**Figure 3:**
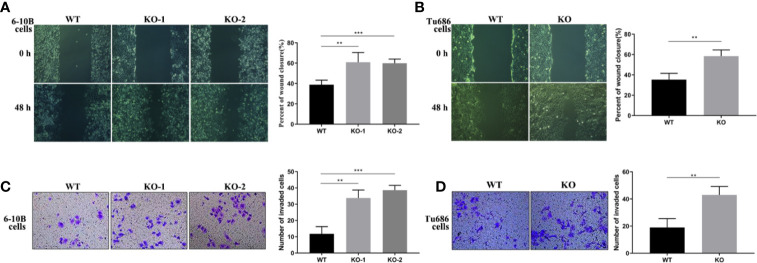
Knockout of PRAS40 increases the invasion and metastasis in HNSCC cell lines. Wounding-healing **(A, B)** and transwell chamber assays **(C, D)** were used to measure the changes following knockout of PRAS40 in 6-10B and Tu686 regarding migration and invasion. It indicates the ability of invasion and metastasis in the knockout of PRAS40 cells is higher than parent cells, respectively. Quantitative data for triplicated experiments are presented in the corresponding right XY plot (**P < 0.01, ***P < 0.005). WT means wildtype. KO means knockout.

To further investigate the function of PRAS40 in the progression of HNSCC cells, wound healing and transwell assays were performed. Wound healing assay demonstrated that cells with PRAS40 knockout migrated more quickly than the wild type cells (**Figure 3**, 6–10B: 37.4 ± 7.1%, 6–10B-KO-1: 58.7 ± 12.7%, 6–10B-KO-2: 58.1 ± 6.6%, p < 0.05; Tu686: 35.3 ± 6.3%, Tu686-KO: 58.4 ± 6.1%, p < 0.05). Similar results were also observed in the number of cells crossing the transwell chamber (6–10B: 56 ± 6, 6–10B-KO-1: 111 ± 11, 6–10B-KO-2: 125 ± 7, p < 0.05; Tu686: 82 ± 8, Tu686-KO: 123 ± 9, p < 0.05), which implied that PRAS40 might inhibit HNSCC cell metastasis.

### The Alteration of Molecular Landscape Induced by PRAS40 in HNSCC

**Figure 4:**
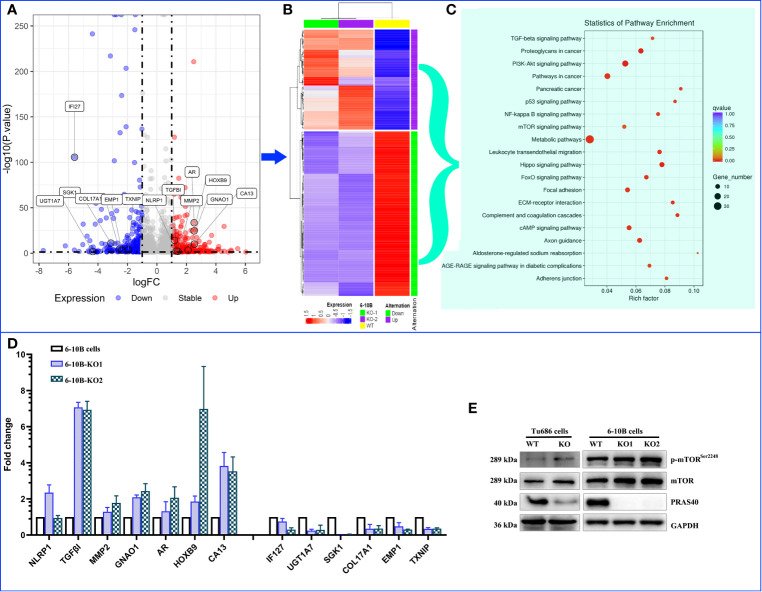
The alteration of molecular landscape induced by PRAS40 in HNSCC. **(A)** Volcano plot shows the logic fold change (logFC on the x-axis) and statistic reliability (p-value on the y-axis) for the global mRNA expression in PRAS40 knockout cells over wildtype cells. The red and blue dots represent up-regulated and down-regulated mRNAs in the PRAS40 knockout cells. Any dot beyond the horizontal dotted line means p <0.05. **(B)** The differential expressed mRNAs from **(A)** were entered to establish a heatmap showing the distinct molecular expression pattern between knockout of PRAS40 cells and their parent cells. **(C)** The top 20 signaling pathways for these differentially expressed genes by the KEGG signaling pathway enrichment analysis. **(D)** qPCR confirms the same changing directions for randomly selected mRNAs from the above RNA-sequencing results(A), all p <0.05. **(E)** Western blot shows the increased phosphorylation of mTOR in PRAS40 knockout cells as compared to their wildtype paraments cells. WT means wildtype. KO means knockout.

To discover the potential molecular mechanism of the above PRAS40-regulating cell malignant behaviors, we employed RNA sequencing in two PRAS40 knockout cell lines as well as their parent cell line. The global mRNA expression profile was compared between wild type and PRAS40 KO cells and overlapped to establish the differential expressions. As shown in **Figure 4A**, a total of 253 genes were differentially expressed in the cells with PRAS40 knockout comparing to wild type, of which 97 mRNAs significantly declined, including IFI27, UGT1A7, SGK1, COL17A1, EMP1, and TXNIP. And there were 156 mRNAs NLRP1, TGF*β*I, MMP2, AR, GNAO1, HOXB9, and CA13 upregulated significantly. These 253 mRNAs constructed the expression of a heatmap showing the distinct molecular expression pattern between PRAS40 knockout cells and their parent cells (**Figure 4B**).

Inputting these 253 genes to KEGG pathway enrichment analysis, we found the top 20 pathways, such as TGF-*β*, PI3K-Akt, P53, mTOR, NF-*k*B signaling pathways, which may shoot the undermined molecular mechanism for PRAS40-regulating cell behaviors (**Figure 4C**). Moreover, 13 of 20 randomly selected mRNAs were validated by qPCR, consistently with our RNA sequencing results (**Figure 4D**). Additionally, the mTOR pathway was activated after the PRAS40 knockout in HNSCC cell lines (**Figures 4E**).

### The Immune Cell Signatures in HNSCC Patients With Different Expression of PRAS40

**Figure 5:**
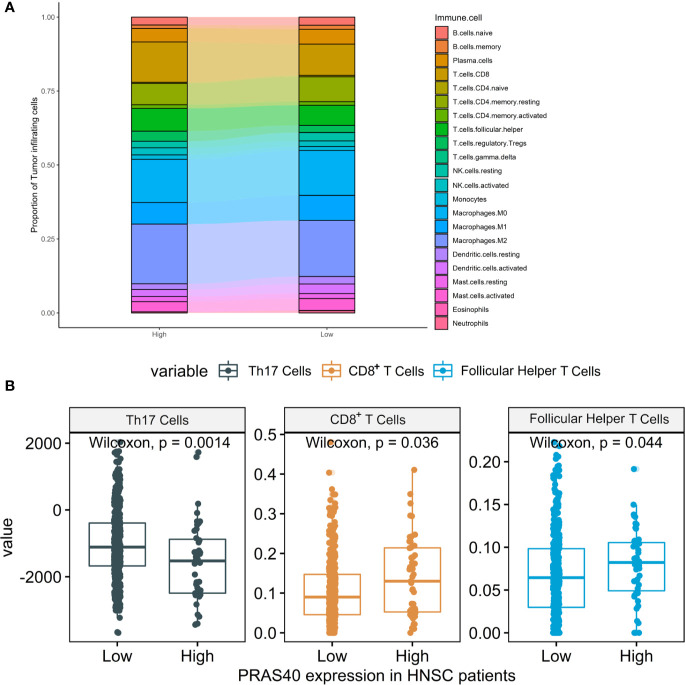
The immune cell signatures in HNSCC patients with different expressions of PRAS40. **(A)** The proportions of 22 tumor-infiltrating immune cells deconvoluted based on the mRNA expression show the landscape alterations of tumor-infiltrating immunity between the HNSC patients with high and low PRAS40 expression. Different color represents a unique type of immune cell, and y-axis value is the mean proportion of immune cell in the group of HNSC patients with different PRAS40 expression. **(B)** High expression of PRAS40 in HNSC patients would present more CD8+ T and T follicular helper cells, but less Th17 cells than the patients with low expression of PRAS40 (all p<0.05).

According to the above results, the downregulation of PRAS40 may involve the TGF-*β* signal pathway, a crucial pathway controlling immune cell fate and functions. Here, we deconvoluted 22 kinds of tumor-infiltrating immune cells based on the mRNA expression in TCGA HNSCC patients with high or low-expression of PRAS40. The comparison of the immune cell landscape was shown in **Figure 5A**, which implied that there were differential proportions of immune cells between patients with high and low expression of PRAS40. For instance, we found that high expression of PRAS40 in HNSCC patients would present more CD8^+^ T and T follicular helper cells, but less Th17 cells than the patients with low expression of PRAS40 (**Figure 5B**).

## Discussion

In the present study, we found that high expression of PRAS40 mRNA is an independent favorable factor for HNSCC patients. To our best knowledge, this is the first report showing the relationship between the PRAS40 expression and survival of HNSCC patients. Previous investigations indicated that PRAS40 could be either an oncogene or a suppressed gene in other types of cancer, such as melanoma, bladder cancer, and cervical cancer (17, 20). Here, our founding stands by PRAS40 might be a suppressor in HNSCC, which keeps pace with the concept that PRAS40 is a potent inhibitor to the mTORC1 activity (26). Decreased expression of PRAS40 would allow the downstream protein of mTORC1, including 4E-BP1 and P70S6K1, to be phosphorylated and activated, which could promote global mRNA transcription and protein synthesis (27). Moreover, our data showed that PRAS40 knockout in several HNSCC cell lines promoted cell colony formation, migration, and invasion. Our results implied that PRAS40 played as a restrainer on HNSCC formation and metastasis, consistent with our clinical data finding that PRAS40 may be a favorable factor for HNSCC prognosis. To ensure the efficiency and accuracy of CRISPR/Cas9 plasmid targeting PRAS40, we had validated the completely lost expression of PRAS40 by DNA sequences and western blot in all our experimental mono-clone cell lines. Therefore, it was highly confident to confirm that reduced PRAS40 expression could predict a worse outcome in HNSCC patients by analyzing both bench and bedside data in our study.

In order to investigate the possible mechanism underlying the regulations of the above biological behaviors by PRAS40 in HNSCC, RNA sequencing technology was applied in our study. We found 253 differentially expressed mRNA when comparing PRAS40-knockout and wild type of HNSCC cell lines. Most of the randomly selected mRNAs were validated by qPCR, making our RNA-seq results more confident to go further analysis. We found that TGF-*β*, PI3K-Akt, P53, mTOR, NF-*k*B signaling pathways are on the top enriched pathways as these 253 differential expressed genes input in KEGG analysis.

The misregulation of TGF-*β* signaling pathway is a vital player in cancer development (28). Our previous work showed that TGF-*β* could promote cell migration and invasion by induction of epithelial-mesenchymal transition in HNSCC cell line (14). Here, we confirmed that transforming growth factor beta-induced (TGFBI) mRNA was remarkably upregulated in HNSCC cells when PRAS40 was knockout. It was reported that TGFBI expressed higher in tumors than adjacent normal specimens within head and neck cancer patients (29). TGFBI may increase tumor metastasis by activating focal adhesion kinase signaling pathway through its binding to integrin *α*V*β*5 (30), which may give some hints about the mechanism of PRAS40- regulating metastasis in HNSCC cell lines.

Numerous publications and our previous work showed the crucial role of the PI3K/Akt pathway in HNSCC initiation and development (31). Interestingly, our unpublished data indicated that PRAS40 could interact with FOXO1 directly, which may lead PRAS40 back to regulate the PI3K/Akt pathway. Additionally, the FOXO pathway was also enriched in our differential expressed mRNAs. However, whether PRAS40 and AKT would competitively bind to FOXO1 still under investigation. If the hypothesis is confirmed in the future, the biological behavior regulated by PRAS40 may be illustrated to some extent that PRAS40 knockout would allow more Akt binds and promotes FOXO1 transcription activity.

Both protein and mRNA expression of the mTORC1 pathway was upregulated after the PRAS40 knockout in our HNSCC cell lines. The first discovered function of PRAS40 was restraining mTORC1 activity (26). When PRAS40 expression was reduced, the over-activation of mTORC1 would not be surprisedly noticed, resulting in tumor cell colony formation and invasion in turn. This mechanism supported our finding that PRAS40 knockout could boost cell colony formation, migration, and invasion in HNSCC cells. Moreover, the NF-*κ*B pathway also was included in the enrichments. That is includes evading growth suppressors, resisting cell death, and enabling replicative immortality. Also, inducing angiogenesis and activating invasion and metastasis acquired during the development of human tumors (32–35). In addition to cancer cell colony formation and survival, NF-*κ*B related inflammation could regulate cancer patients’ local and systemic immunity (36, 37). It is unclear how much the NF-*κ*B pathway plays in the PRAS40-regulating HNSCC cell growth and metastasis. However, our previous work indicated that PRAS40 could directly interact with P65 and promote NF-*κ*B transcriptional activity (17), including various types of downstream targets such as pro-apoptotic genes (Fas, TP53, P21, and DR4/5) and anti-apoptotic genes (Bcl2, XIAP).

The investigation of the complexity and diversity of tumor immune microenvironment may help us understand the potential phenotypes. Our study showed that the HNSC patients with high PRAS40 expression would present more CD8+ T and T follicular helper (Tfh) cells but less Th17 cells in tumor samples than the patients with low PRAS40 expression. Tfh cell is a distinct subpopulation of CD4^+^ T cells that differentiates in secondary lymphoid organ germinal centers and plays critical roles in helping antigen-specific B cells generate antibodies (38). Tfh cells were also identified as residents in the tertiary lymphoid structure within tumor specimens (39). Th17 cells (characterized by high secretion of IL-17) differentiated from T helper lymphocytes participate in antimicrobial immunity at mucosal and epithelial barriers (40). Th17 cells’ prevalence was accumulated in peripheral blood, tumors, and drain lymph nodes of HNSCC patients that may promote tumor growth by inducing angiogenesis (*via* IL-17) and exerting themselves immunosuppressive functions (41–43). From an immune perspective, the high proportions of CD8+ T and Tfh cells and low proportion of Th17 cells may be the reasons for the favorable survival time in the group of HNSCC patients with high PRAS40 expression. Regarding the possible mechanism of different distributions of CD8^+^ T, Tfh, and Th17 cells in HNSCC patients with high or low expressed PRAS40, there are some clues from the molecular landscape alternations in PRAS40 knockout HNSCC cells: the overall effects of TGF-*β*, PI3K-Akt, NF-*κ*B, and mTORC1 signaling.

All in all, this is the first report to show that the PRAS40 expression would be an independent prognosis factor in HNSCC patients. Furtherly, PRAS40 was confirmed as a suppressor of cell colony formation, migration, and invasion in HNSCC cell lines. Finally, we described a molecular landscape alternation in PRAS40 knockout HNSCC cell lines, which was validated in this study and partially our previous work. The differential distributions of CD8+ T cells, Tfh cells, and Th17 cells associating with the prognosis of HNSCC patients with high or low expression of PRAS40 solidified our conclusion that PRAS40 could be a tumor suppressor in HNSCC patients.

## Data Availability Statement

The original contributions presented in the study are publicly available. This data can be found here: https://www.ncbi.nlm.nih.gov/geo/query/acc.cgi?acc=GSE163613.

## Author Contributions

XZ, GZ, and YL conceived and designed the experiments. GC and ZL performed most of the cell experiments. CC did PCR and data analysis. WZ, YQ, JL, and GL did TCGA analysis. GZ and X Z wrote the paper. DH, QT contributed to the establishment of cell lines with PRAS40 knockout. HH, LS, and JW reviewed the draft. All authors contributed to the article and approved the submitted version.

## Funding

This research was supported by the Project of Hunan Health Commission (B2019165), National Natural Science Foundation of China (Nos. 81874133, 81602389 and 81772903), Natural Science Foundation of Hunan Province (Nos. 2020JJ4827, 2018JJ2630, 2017JJ3456, and 2018JJ3317), the Huxiang Youth Talent Project (No. 2018RS3024), and National Key Research and Development Project (Nos. 2020YFC1316900 and 2020YFC1316901).

## Conflict of Interest

The authors declare that the research was conducted in the absence of any commercial or financial relationships that could be construed as a potential conflict of interest.

## Supplementary Material

Supplementary Figure 1 The expression of PRAS40 in mono-colonies cells with PRAS40-knockout. The expression of PRAS40 was confirmed in eight cell lines of 6-10B and six cell lines of Tu686. It showed the silencing expression of PRAS40 in 6-10B and Tu686 KO cells as compared to the corresponding parent cells, respectively. Especially 6-10B KO-1, 6-10B KO-2, and Tu686 KO1 (named Tu686-KO in **Figures 2**–**4**) cell lines, which are selected for the future cell culture and experiments.

## Abbreviations

TCGA, The Cancer Genome Atlas; NCBI, National Center for Biotechnology Information; GO, gene ontology; KEGG, Kyoto Encyclopedia of Genes and Genomes; DEGs, differential expression genes; ANOVA, analysis of variance; PRAS40, Prolin-rich Akt substrate of 40 kDa; HNSCC, head and neck squamous cell carcinoma; CCDS, consensus coding sequence; TGFBI, growth factor beta-induced; Tfh, T follicular helper; OS, overall survival.
