# Supplementary figures and images for: The Molecular Landscape and Biological Alterations Induced by PRAS40-Knockout in Head and Neck Squamous Cell Carcinoma
Source: Front Oncol. 2021 Jan 8;10:565669. doi: 10.3389/fonc.2020.565669 (PMC7821427; doi:10.3389/fonc.2020.565669)

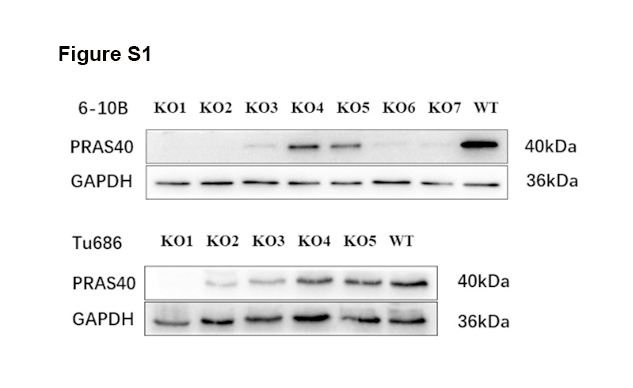

Supplement: Supplementary Figure 1 — The expression of PRAS40 in mono-colonies cells with PRAS40-knockout. The expression of PRAS40 was confirmed in eight cell lines of 6-10B and six cell lines of Tu686. It showed the silencing expression of PRAS40 in 6-10B and Tu686 KO cells as compared to the corresponding parent cells, respectively. Especially 6-10B KO-1, 6-10B KO-2, and Tu686 KO1 (named Tu686-KO in Figures 2 – 4 ) cell lines, which are selected for the future cell culture and experiments. [file Image_1.tif]
