# Supplementary material for: The Molecular Landscape and Biological Alterations Induced by PRAS40-Knockout in Head and Neck Squamous Cell Carcinoma
Source: Front Oncol. 2021 Jan 8;10:565669. doi: 10.3389/fonc.2020.565669 (PMC7821427; doi:10.3389/fonc.2020.565669)
Supplement: Supplementary file 2 [file DataSheet_1.docx]

| **Gene** | **sequence** |
| --- | --- |
| PRAS40 sgRNA1 | TTGCCTCCACGACATCGCAC NGG |
| PRAS40 sgRNA2 | CGCCAGGGCTCCTCGACCAT NGG |
| PRAS40 sgRNA3 | CCGCAGGGCGCGGATGGCGT NGG |
| PRAS40-1 Forward | 5’-CCT GCT CTG TGT CCT TGT GC -3’ |
| PRAS40-1 Reverse | 5’-TGA CTT GGG GCA GGA GGA AT-3’ |
| PRAS40-2 Forward | 5’-ATC TGT TCG GGA CAA TGT GAC TC-3’ |
| PRAS40-2 Reverse | 5’-GCT CGT TCG GCT TTA TTG TAC TC -3’ |
| GAPDH Forward | 5’-TCC AAA ATC AAG TGG GGC GA-3’ |
| GAPDH Reverse | 5’-AGT AGA GGC AGG GAT GAT GT-3’ |

**Table S1.** The sequence of sgRNA and primers
